# MR-Guided Radiotherapy in Oesophageal Cancer: From Principles to Practice—A Narrative Review

**DOI:** 10.3390/curroncol33010034

**Published:** 2026-01-08

**Authors:** Su Chen Fong, Eddie Lau, David S. Liu, Niall C. Tebbutt, Richard Khor, Trevor Leong, David Williams, Sergio Uribe, Sweet Ping Ng

**Affiliations:** 1 Department of Radiation Oncology, Austin Hospital, Heidelberg, VIC 3084, Australia; 2 Department of Radiation Oncology, Peter MacCallum Cancer Centre, Melbourne, VIC 3000, Australia; 3 Department of Medical Imaging and Radiation Sciences, School of Primary and Allied Health Care, Faculty of Medicine, Nursing and Health Sciences, Monash University, Melbourne, VIC 3800, Australia; 4 Department of Radiology and Molecular Imaging Therapy, Austin Health, Heidelberg, VIC 3084, Australia; eddie.lau@austin.org.au; 5 Department of Surgery, University of Melbourne, Melbourne, VIC 3010, Australia; 6 Upper Gastrointestinal Surgery Unit, Division of Surgery, Anaesthesia and Procedural Medicine, Austin Health, Heidelberg, VIC 3084, Australia; 7 Division of Cancer Surgery, Peter MacCallum Cancer Centre, Melbourne, VIC 3000, Australia; 8 Victorian Interventional Research and Trials Unit, Department of Surgery, University of Melbourne, Austin Precinct, Heidelberg, VIC 3010, Australia; 9 Department of Medical Oncology, Austin Health, Heidelberg, VIC 3084, Australia; 10 Sir Peter MacCallum Department of Oncology, University of Melbourne, Melbourne, VIC 3010, Australia; 11 Department of Pathology, Austin Health, Heidelberg, VIC 3084, Australia; 12 Olivia Newton-John Cancer Wellness and Research Institute, Heidelberg, VIC 3084, Australia; 13 School of Cancer Medicine, La Trobe University, Bundoora, VIC 3086, Australia

**Keywords:** oesophageal cancer, tumour delineation, functional MRI, diffusion-weighted imaging, MR-guided radiotherapy, MR-Linac, adaptive radiotherapy, motion management, personalised radiotherapy

## Abstract

Oesophageal cancer remains a significant global health burden with poor survival outcomes despite multimodal treatment. Recent advances in magnetic resonance imaging (MRI) have opened opportunities to improve radiotherapy delivery. This review examines the role of MRI and MR-guided radiotherapy (MRgRT) in oesophageal cancer, focusing on applications in staging, treatment planning, and response assessment, with particular emphasis on magnetic resonance linear accelerator (MR-Linac)-based delivery. Compared to computed tomography (CT), MRI offers superior soft-tissue contrast, enabling more accurate tumour delineation and the potential for reduced treatment margins. Real-time MR imaging during treatment can facilitate motion management, while daily adaptive planning can accommodate anatomical changes throughout the treatment course. Functional MRI sequences, including diffusion-weighted and dynamic contrast-enhanced imaging, offer quantitative data for treatment response monitoring. Early clinical and dosimetric studies demonstrate that MRgRT can significantly reduce radiation dose to critical organs while maintaining target coverage. However, clinical evidence for MRgRT in oesophageal cancer is limited to small early-phase studies, with no phase II/III trials demonstrating improvements in survival, toxicity, or patient-reported outcomes. Long-term clinical benefits and cost-effectiveness remain unproven, highlighting the need for prospective outcome-focused studies to define the role for MRgRT within multimodality treatment pathways.

## Simple Summary

Oesophageal cancer is an aggressive disease with poor survival despite advances in combined chemotherapy, radiotherapy, and surgery. Radiotherapy plays a crucial role, but its precision is limited by the inability of conventional imaging such as computed tomography (CT) and positron emission tomography (PET) to clearly visualise the tumour and surrounding organs. Magnetic resonance imaging (MRI) provides excellent soft-tissue detail and can be integrated directly with a radiotherapy system, known as MR-guided radiotherapy (MRgRT). This technology enables daily imaging of the tumour during treatment, real-time motion tracking, and on-the-spot plan adjustments to account for anatomical changes. It also offers new ways to monitor tumour response using functional MRI sequences that reflect biological activity. While early clinical studies demonstrate improved accuracy and reduced radiation to the heart and lungs, wider adoption will depend on overcoming technical, workflow, and access challenges. MRgRT represents a promising step toward personalised, adaptive radiotherapy for oesophageal cancer.

## 1. Introduction

Oesophageal cancer remains an aggressive malignancy, with most patients presenting with locally advanced disease and poor survival outcomes despite multimodality therapy [1]. Radiotherapy is a key component of curative-intent management, including definitive chemoradiotherapy and neoadjuvant treatment prior to surgery. Accurate imaging is therefore critical across the treatment pathway, from initial staging and treatment selection to radiotherapy planning, delivery, and response assessment.

Current staging and planning rely on endoscopic ultrasound (EUS), computed tomography (CT), and positron emission tomography (PET). Although these modalities provide complementary information, soft-tissue contrast remains suboptimal, and they lack real-time tumour visualisation. Magnetic resonance imaging (MRI) offers potential advantages with its superior soft-tissue contrast and multiparametric data that can improve the assessment of tumours. Advances in MRI acquisition techniques have increased its feasibility in oesophageal cancer, enabling applications beyond diagnosis, including radiotherapy planning and treatment response evaluation [2,3]. Integration of MRI into linear accelerator systems has enabled magnetic resonance-guided radiotherapy (MRgRT), allowing daily soft-tissue visualisation, real-time motion management, and online adaptive planning.

This narrative review examines current evidence for MRI and MRgRT in oesophageal cancer, focusing on staging, radiotherapy planning, and treatment response monitoring. This review also highlights the potential for adaptive and personalised radiotherapy, as well as current limitations and the need for prospective clinical validation.

## 2. Methods

To identify relevant literature, we performed a comprehensive search of PubMed, Embase, and the Cochrane Library databases for studies published between 2000 and August 2025. Search terms combined oesophageal and gastroesophageal junction disease descriptors (“oesophag*”, “esophag*”, “gastroesophageal junction”, “gastro-oesophageal”), radiotherapy-related terms (“radiotherapy”, “chemoradiotherapy”, “image-guided radiotherapy”, “adaptive radiotherapy”, “online adaptive”), MRI-related terms (“magnetic resonance imaging”, “MR simulation”, “MR-Linac”, “MRI-guided radiotherapy”), and functional MRI terms (“diffusion-weighted imaging”, “apparent diffusion coefficient”, “dynamic contrast-enhanced MRI”, “perfusion MRI”).

Reference lists of reviews were screened for additional sources. Priority was given to systematic reviews, meta-analyses, and prospective clinical trials. Due to the limited amount of prospective MRgRT data, feasibility studies, dosimetric comparisons, and relevant retrospective series were also included. Studies were restricted to those published in English. Study selection focused on MRI diagnostic accuracy, functional MRI, and technical or clinical implementation of MRgRT in oesophageal cancer.

## 3. Utility of MRI in Oesophageal Cancer Staging

### 3.1. Current Standard Limitations

Accurate staging of oesophageal cancer is essential to guide treatment and prognosis. EUS remains the most accurate test for locoregional assessment. Meta-analyses report T-staging pooled sensitivities and specificities of 81.6% and 99.4% for T1 disease, and 92.4% and 97.4% for T4 disease, with overall T-staging accuracy of 79% and N-staging of 71% [4]. The addition of fine-needle aspiration markedly improves nodal assessment, raising sensitivity to 97% and specificity to 96% [4]. However, accuracy is operator-dependent, technically unfeasible in up to 30% of patients with stenotic tumours, and less reliable in distinguishing T1/T2, which is clinically important as ≥T2 stages usually require multimodality therapy [5].

CT is useful for evaluating distant metastases and resectability but has limited accuracy in determining cranio-caudal extent, with T-staging as low as 43% and nodal sensitivity of 30% to 60% [3]. 18F-FDG PET complements CT by improving delineation of cranio-caudal tumour extent and detecting distant metastases [3]. PET/CT also provides high specificity (96%) for nodal disease, but the sensitivity is modest (62%), meaning micrometastases may be missed [4,5]. Inflammatory conditions can also cause false positives, lowering specificity.

### 3.2. Staging MRI Performance

**Figure 1:**
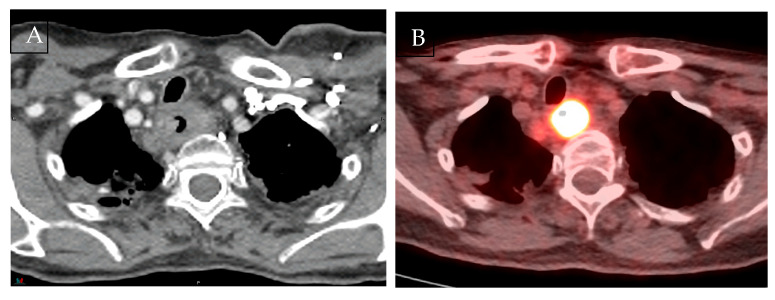

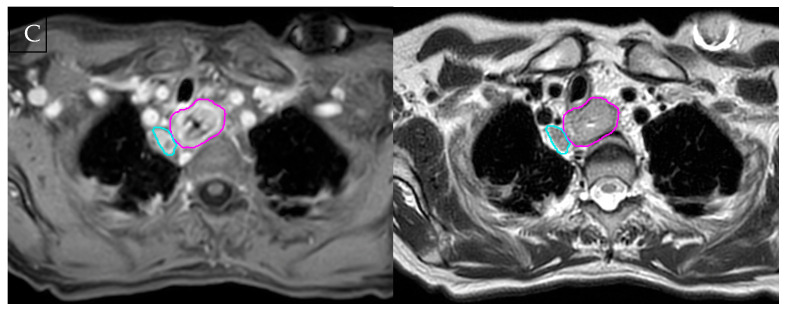
Multimodality imaging comparison in oesophageal cancer. (**A**) Axial contrast-enhanced CT at the thoracic inlet. (**B**) 18F-FDG PET/CT at the same level shows avid uptake consistent with a metabolically active tumour, but with limited spatial resolution, especially in the adjacent node. (**C**) T1 and T2-weighted MRI with gross tumour volume (pink) and suspicious node (blue) contoured. Although the tumour abuts the trachea, MRI clearly demonstrates an intact interface and preserved tracheal cartilage. All images were acquired on the authors’ institutional MR-Linac and are presented for illustration only.

With better soft-tissue contrast, MRI can visualise oesophageal wall layers and tumour–organ interfaces, which is particularly relevant for suspected T4 disease. Unlike CT or PET, MRI may help exclude true invasion by demonstrating preserved anatomical planes, as illustrated in Figure 1. Early small series summarised by van Rossum et al. have reported very high accuracy for identifying T4 disease, including reports of 100% accuracy [2]. Riddel et al. reported an 83% accuracy rate in distinguishing between T2 and T3 stages when compared to histological examination [6]. A meta-analysis by Lee et al. examining 20 studies found MRI had a pooled sensitivity and specificity of 86% for distinguishing ≥T3 from ≤T2 tumours. However, specificity was lower for T1 disease, increasing the likelihood of false positives [7].

For N-staging, MRI shows a pooled sensitivity of 71% (95% CI: 60–80) and specificity of 72% (95% CI: 64–79) for differentiating node-negative from node-positive disease [7]. This can be improved using gadolinium-enhanced and nanoparticle-enhanced MRI. Gadolinium-enhanced 1.5T MRI can achieve a sensitivity up to 100% and a specificity of 78% [8]. Nanoparticle-enhanced MRI further improves specificity to 95.4% while maintaining high sensitivity in small cohorts [9,10]. These particles, such as superparamagnetic iron oxide (SPIO) and ultrasmall SPIO (USPIO), act as negative contrast agents that can be used to identify non-enlarged metastatic lymph nodes. The ECG-triggered Short Tau Inversion Recovery sequence (fat suppression technique) further enhances the detection of pathological lymph nodes (sensitivity 81.3%, specificity 98.3%) [11]. For M-staging, integrated 18F-FDG PET/MRI showed improved sensitivity (78.3%) compared to 18F-FDG PET/CT (52.2%) [12].

Recent studies evaluated whether artificial intelligence and radiomics can further improve staging accuracy across modalities. CT- and PET-based radiomics improved T- and N-staging discrimination by quantifying intratumoral heterogeneity beyond visual assessment alone [13,14]. While AI improves feature extraction across these modalities, it cannot overcome the intrinsic soft-tissue limitations of CT or PET, nor can it provide cross-sectional anatomical detail from endoscopy. As such, AI augmentation of other modalities is unlikely to fully replicate the anatomical and biological information obtainable from MRI. Preliminary machine learning and MRI-based radiomics studies similarly report encouraging results (AUC 0.71-0.85), although these remain retrospective, single-centre studies and lack external validation [15,16].

### 3.3. Technical Advances

Technical refinements have improved MRI feasibility for oesophageal imaging, including respiratory gating, cardiac gating, and free-breathing radial acquisition techniques such as r-VIBE, which allow shorter acquisition times [17,18,19]. The addition of cine sequences further increases diagnostic confidence in T-staging, with disruption of oesophageal peristalsis indicating muscle invasion [20,21]. Together, these approaches mitigate motion artefacts and enhance diagnostic accuracy, particularly when assessing tumour–organ interfaces. Contrast-enhanced r-VIBE using 3.0T MRI has also demonstrated higher accuracy compared to EUS in staging advanced tumours, with good inter-reader agreement [22]. While ultrahigh-field MRI (4.7T and 7.0T) demonstrated up to 100% accuracy of T- and N-staging in ex vivo studies, its clinical applicability remains exploratory [23,24,25]. The challenge is to translate these improvements into meaningful diagnostic or therapeutic outcomes in clinical practice.

## 4. MRI in Radiotherapy Treatment Planning

### 4.1. Tumour Visualisation and Delineation

MRI offers significant advantages for tumour visualisation and delineation during radiotherapy planning. Although MRI is routinely used for radiotherapy treatment planning across several anatomical sites, including the central nervous system, head and neck, prostate, and cervix [26], its routine clinical adoption in oesophageal cancer has been restricted by accessibility and workflow-related challenges, though this is evolving. The clinical rationale for its integration is compelling, given the limitations of current standard imaging.

A standard radiotherapy workflow typically relies on CT-based planning fused with FDG-PET/CT and correlated with endoscopic findings. However, the accuracy of tumour delineation is frequently compromised by the limited spatial resolution of PET and suboptimal soft-tissue contrast, particularly for determining the longitudinal tumour extent [27,28].

**Figure 2:**
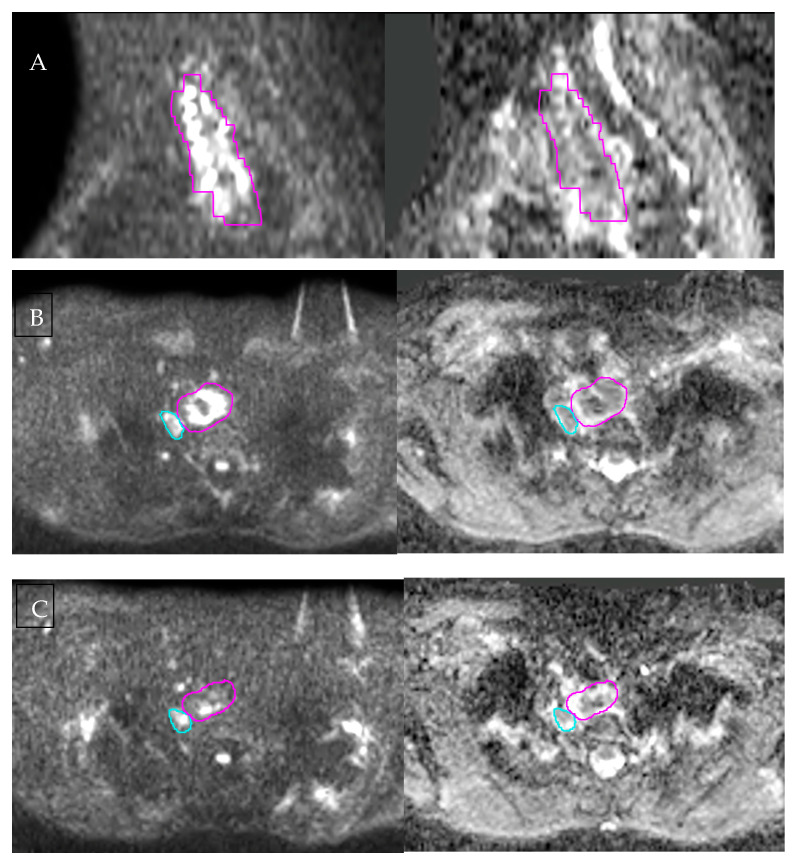
Diffusion-weighted MRI for baseline tumour characterisation and early treatment-related change. (**A**) Baseline sagittal DWI and corresponding ADC map illustrate the craniocaudal extent of the primary oesophageal tumour. (**B**) Baseline axial DWI and ADC demonstrate marked diffusion restriction within the tumour, with the gross tumour volume (pink) and a suspicious lymph node (blue) contoured. (**C**) Mid-treatment axial DWI and ADC acquired after 13 fractions of chemoradiotherapy show reduced diffusion restriction and increased ADC values. All images were acquired on the authors’ institutional MR-Linac and are presented for illustration only. Figure 1 and Figure 2 are derived from the same imaging dataset.

Several complementary MRI sequences contribute to improved tumour visualisation for radiotherapy planning, with the T2-weighted sequence defining wall anatomy and diffusion-weighted imaging assisting in distinguishing tumour from surrounding oedema or inflammation. An example of diffusion-restricted tumour extent on baseline DWI is shown in Figure 2A.

Multiparametric MRI combining T1-weighted, fat-suppressed T2-weighted, and DWI significantly improves the accuracy of tumour measurement, and is particularly useful for gastroesophageal junction (GEJ) tumours [27,29]. Furthermore, MRI provides a more consistent and reproducible target delineation, which reduces intra-observer and inter-observer variability [26,30,31]. Hou et al. demonstrated that MRI-based delineation showed minimal discrepancies in gross tumour volume (GTV)–length measurements compared to pathology (differences of 0.54 mm using diffusion-sensitive gradient b-values of 400–800 s/mm^2^), while CT-based delineation exhibited significantly larger differences (3.63 mm) [32].

By reducing the uncertainty in defining the GTV and volumes of surrounding critical organs, MRI-based delineation may allow for smaller treatment margins and more precise radiation delivery. Implementation challenges remain, including the need for specialised training and consensus on MRI-specific contouring guidelines. Emerging developments in automated MRI-based contouring show promise, potentially streamlining workflow while maintaining or improving accuracy [33].

### 4.2. Magnetic Resonance Simulation (MR-Sim) in Radiation Oncology

The standard radiotherapy workflow utilises CT as the primary planning modality, which provides electron density information required for dose calculation. However, registration uncertainties arise when diagnostic MRI is co-registered with CT due to differences in image acquisition parameters, slice orientation, patient positioning, and immobilisation. Diagnostic MRI frequently uses angulated planes optimised for organ assessment, whereas radiotherapy imaging requires orthogonal, thin-slice acquisition planes with geometric correction to ensure spatial accuracy.

MR-Sim addresses these limitations using dedicated protocols and specialised hardware such as MRI-compatible immobilisation devices, flat tabletops, coil bridges, wider bores, and laser positioning systems [34]. This improves the reproducibility of patient setup and streamlines accurate registration with planning CT datasets.

For MR-Linac workflows and radiotherapy MRI, geometric fidelity is therefore a critical requirement. Spatial distortion from gradient non-linearity, B0 inhomogeneity, and susceptibility effects is particularly relevant in the thorax near air–tissue interfaces and should be characterised and managed within the radiotherapy field of view in treatment position, consistent with MR simulation quality-assurance guidance such as AAPM TG 284 [35]. In thoracic MR-Linac workflows, the magnetic field also alters secondary electron transport and can cause interface dose perturbations known as the electron return effect [36]. It can also produce out-of-field surface dose via electron streaming, including coil-related streaming in some configurations [37,38]. These effects should be modelled in the treatment planning system and mitigated through appropriate beam arrangement and coil positioning with minimisation of air gaps, with targeted local verification where indicated [36,38].

CT-related image registration uncertainties may be eliminated with MRI-only planning [39,40]. However, the lack of electron density data must be addressed. Synthetic CT methods, including atlas-based approaches and machine learning algorithms, have demonstrated promising results, with dosimetric accuracy approaching that of conventional CT-based planning [40,41].

MR-sim protocols also enable simultaneous acquisition of anatomical and functional sequences in treatment position, creating opportunities for offline adaptive strategies and longitudinal assessment of anatomical and biological information during treatment.

## 5. Functional Imaging and Response Assessment

The ability of standard imaging modalities to distinguish between residual disease and post-treatment inflammation is somewhat limited. This difficulty was highlighted by the preSANO trial, which found that conventional surveillance methods combining EUS with bite-on-bite biopsies and FDG-PET/CT missed up to 31% of significant residual disease (Chirieac modified TRG3 or TRG4) [42].

Before morphologic changes become evident, functional MRI techniques like DWI, DCE-MRI, and intravoxel incoherent motion (IVIM) can provide non-invasive ways to evaluate cellularity, perfusion, and microstructural changes.

### 5.1. Diffusion-Weighted Imaging (DWI) and Apparent Diffusion Coefficient (ADC)

DWI measures the Brownian motion of water molecules within tissue and provides indirect information on tissue cellularity and microstructure. Malignant tissues typically show restricted diffusion due to high cellular density and reduced extracellular space, resulting in high signal intensity on high-b-value DIW and low values on corresponding ADC maps. At baseline, multiple studies have demonstrated that lower ADC values are associated with more-advanced disease stage, poorer differentiation, and an increased likelihood of nodal involvement [43,44]. Beyond baseline tumour characterisation, serial ADC measurements during treatment are promising biomarkers for early treatment response. During treatment, diffusion restriction diminishes, and ADC values rise due to tumour necrosis and decreased cellularity. This temporal change is illustrated in Figure 2, demonstrating changes from baseline and mid-treatment imaging.

Importantly, dynamic ADC changes (ΔADCs) during treatment appear to provide greater prognostic value than static baseline measurements in predicting response to treatment [45,46]. A recent meta-analysis of 21 studies (1128 patients) reported pooled sensitivity, specificity, and AUC of 0.82, 0.81, and 0.88, respectively, for DWI in predicting pathological response to concurrent chemoradiotherapy [45]. Significant increases in ADC as early as two to three weeks into treatment have been correlated with pathological complete response (pCR), whereas a lack of early change is linked to a higher recurrence risk [47,48].

### 5.2. Dynamic Contrast-Enhanced MRI (DCE-MRI), Intravoxel Incoherent Motion (IVIM), and Hypoxia-Sensitive Functional MRI

DCE-MRI provides quantitative metrics reflecting vascular permeability and perfusion, such as K_trans_ (transfer constant) and K_ep_ (rate constant). A reduction in these parameters has been correlated with pCR and is predictive of treatment response [49,50,51]. In a pilot study by Heethuis et al., all patients who achieved pCR exhibited at least a 25% reduction in the gadolinium uptake AUC during treatment [49].

IVIM further differentiates true molecular diffusion (D) from perfusion-related diffusion (D*) and perfusion fraction (f). Prospective studies have found that baseline IVIM metrics correlate with tumour regression and nodal downstaging [52,53]. These sequences, when used in conjunction with DWI and T2-weighted imaging, may offer a more comprehensive understanding of tumour microenvironment and treatment response.

The standardisation of quantitative imaging techniques is a challenge, but recent technical validation studies on hybrid MR-Linac systems have demonstrated that DWI and DCE-MRI parameters can be acquired with high accuracy (<5% error) and good repeatability, supporting the feasibility of integrating serial functional imaging into MRgRT workflows [54].

Tumour hypoxia can influence radioresistance, and MRI offers non-invasive information about radiobiology. Techniques such as blood oxygen level-dependent (BOLD-MRI) imaging, tissue oxygen level-dependent (TOLD-MRI) imaging, and fluorine-19 MRI can evaluate changes in relaxation properties caused by tissue oxygen content. Early work in oesophageal squamous cell carcinoma has shown that lower pre-treatment T2* values are associated with more advanced tumour stage [55]. Evidence in oesophageal cancer is limited, and most validation comes from other tumour sites, where oxygen-enhanced MRI and BOLD/TOLD MRI can estimate hypoxic fractions and have shown potential for predicting radiotherapy outcomes [56,57].

Among functional MRI techniques, DWI and ADC metrics currently have the strongest evidence base, whereas DCE-MRI and IVIM remain investigational, and hypoxia-sensitive MRI techniques are exploratory in oesophageal cancers.

### 5.3. Multimodal Integration: PET/MRI, Radiomics, and Emerging Techniques

Combining MRI-derived biomarkers with metabolic imaging from FDG-PET can improve the predictive accuracy of treatment response. PET metrics such as ΔSUV_max, reflecting changes in the maximum standardised uptake value, complement diffusion-driven changes on MRI, and when integrated with ΔADC from hybrid PET/MRI, these parameters can increase pCR prediction accuracy to as high as 89% [45,58,59]. The modalities, along with circulating tumour DNA (ctDNA) and blood-based biomarkers, are being studied in trials such as PRIDE to build integrated predictive models [60].

Radiomics enables the high-throughput extraction of imaging features to quantitatively characterise tumour phenotype. In oesophageal cancer, MRI-based radiomics can achieve high diagnostic performance (AUC 0.89-0.97) for predicting pCR [50]. Delta-radiomics, which tracks temporal changes in imaging features during treatment, provides additional prognostic information and may improve the stratification of responders versus non-responders [52]. Multimodal radiomics incorporating both MRI and CT features has also shown improved survival risk stratification after definitive chemoradiotherapy in recent cohorts [61].

Although still investigational, emerging MRI techniques may add complementary biological information. Chemical exchange saturation transfer imaging is sensitive to tissue microenvironmental changes, and MR spectroscopy may reflect metabolic alterations in oesophageal tumours [62]. These approaches require further validation but highlight the potential for expanded functional imaging beyond standard diffusion and perfusion sequences.

Early machine learning and deep learning studies that combine diffusion, perfusion, and multimodal imaging features have shown promising accuracy for predicting pathological response [16,50,61]. Most work remains retrospective and single-centre, and reproducibility is limited by variation in MRI acquisition and radiomic feature extraction, so larger datasets and standardised protocols will be important for broader clinical translation.

## 6. MR-Guided Adaptive Radiotherapy

### 6.1. Treatment Verification and Motion Management

Anatomical variations during treatment present significant challenges for accurate radiation delivery. Cone-beam CT (CBCT) is typically used for treatment verification in image-guided radiation therapy. Although CBCT visualises bone well, it has limitations in soft-tissue contrast compared to MRI [63]. Consequently, oesophageal radiotherapy typically relies on matching vertebral alignment rather than direct tumour matching, necessitating larger planning target volume (PTV) margins of 8–10 mm to account for positioning uncertainties [64]. Soft-tissue matching with fiducial markers allows smaller anisotropic margins of 5.6–6.9 mm, but similar margins are achievable with MR guidance without additional fiducials [65].

There are two commercial platforms available for clinical use, combining real-time MRI with linear accelerator capabilities. The ViewRay MRIdian (0.35T) emphasises continuous intrafraction tracking and gating, while the Elekta Unity (1.5T) focuses on structured pre-treatment adaptation using the Adapt-to-Position and Adapt-to-Shape workflows.

Oesophageal tumours are subjected to significant motion from peristalsis, and respiratory and cardiac activity. Cine-MRI studies demonstrate mean peak-to-peak displacements of 12.7–13.3 mm in the cranio-caudal direction, with smaller movements in the anterior–posterior (3.8–4.9 mm) and lateral (2.7 mm) directions [66,67]. Distal tumours near the junction and cardiac regions display the greatest motion (anteroposterior and craniocaudally), often resulting in insufficient target coverage [20,68].

Conventional passive approaches utilise internal target volumes (ITVs) from 4D-CT to encompass the full range of tumour motion, often requiring significant margins up to 18 mm in the superior–inferior direction [65,69]. Active strategies, such as respiratory gating and real-time tracking, can significantly reduce treatment volumes, but surrogate markers (fiducials, surface monitoring, spirometry) are frequently relied on to accurately represent tumour position [70].

MR-Linac enables continuous soft-tissue visualisation during treatment delivery and supports online adaptation based on visible gross tumour rather than surrogate structures. This allows very tight margins in selected upper abdominal and thoracic stereotactic treatments, with GTV to PTV expansions as small as 3 mm being technically achievable [71]. However, applying such margins to conventionally fractionated oesophageal radiotherapy requires caution, as oesophageal cancers frequently demonstrate longitudinal submucosal microscopic spread, which defines the clinical target volume (CTV). MRgRT therefore justifies reducing setup and motion margins; however, margin reduction should be confined to the CTV to PTV expansion. Daily online adaptive MR-Linac radiotherapy using smaller 6 mm CTV to PTV margins demonstrated significantly improved geometric target coverage while reducing doses to surrounding normal tissues compared with CBCT-guided approaches [57,58]. For GEJ tumours, respiratory-gated MR-Linac plans with 3 mm CTV to PTV margins have considerably decreased cardiac doses through mean PTV reduction from 1275 cm^3^ to 689 cm^3^ [72].

In addition to geometric adaptation, MR-Linac allows ongoing assessment of tumour response throughout treatment. Oesophageal tumours often demonstrate volume reduction (~23%) by the fourth week of treatment, allowing for progressive margin reduction and dynamic normal tissue sparing [73,74]. MR-guided therapy can also correct for tumour drift during treatment, which averages 1.5 mm (range up to 11.6 mm) [66]. Implementation of anisotropic PTV margins of 2–3 mm for upper abdominal tumours using combined gating and drift correction allows substantial treatment volume reduction without compromising coverage [75].

Artificial intelligence technologies are enhancing MR-guided workflows through automated segmentation and motion tracking. AI-based segmentation significantly improves workflow efficiency, enabling adaptive treatments to achieve comparable speed while maintaining dosimetric advantage [76]. Deep learning algorithms for motion tracking have demonstrated sub-millimetre accuracy in motion prediction across thoracic, abdominal, and pelvic regions, with processing speeds suitable for real-time applications [77]. Future advancements may eliminate the need for uncomfortable setup devices and breath-hold treatments [78,79].

### 6.2. Personalised Treatment Opportunities

Marked heterogeneity in treatment response provides a compelling rationale for personalised approaches. pCR rates following chemoradiotherapy range from 16 to 40%, reflecting differences in tumour biology and histology [80,81]. Despite trials showing histology-dependent responses, conventionally fractionated radiotherapy doses are applied uniformly across patients [82,83]. High rates of isolated local recurrences occurring within the GTV (90%) suggest that current dosing may be suboptimal for a subset of patients [84].

While meta-analyses support a dose–response relationship for doses ≥ 60 Gy, particularly for squamous cell carcinoma, clinical evidence remains mixed [83,85]. The ARTDECO trial did not demonstrate improved local control with escalation above 50.4 Gy, and other studies reported increased toxicity beyond 66 Gy [83,85,86]. However, PET/CT-guided dose escalation targeting metabolically active subvolumes up to 72 Gy has shown feasibility and safety [87,88]. Ongoing trials in Germany (NCT01348217 and NCT02556762) and the United Kingdom (NCT02741856) continue to investigate optimal dosing strategies.

MR-Linac could accelerate biologically guided adaptive radiotherapy by acquiring functional data during treatment [89]. Dynamic ADC and perfusion changes, combined with FDG-PET metrics (such as ΔSUV), could guide targeted dose redistribution or early changes in systemic therapy for poor responders [58]. The added value of incorporating hypoxia-mapping MRI into the response-adapted oesophageal cancer treatment paradigm remains investigational.

Organ-sparing approaches are being evaluated in several prospective trials. In the phase 3 SANO trial, 35% of participants managed with active surveillance after achieving a clinical complete response to neoadjuvant chemoradiotherapy avoided oesophagectomy while maintaining non-inferior 2-year overall survival compared with immediate surgery (74% vs. 71%) [90]. This strategy relies on an intensive assessment protocol incorporating endoscopy with bite-on-bite biopsies, EUS with fine needle aspiration, and FDG-PET/CT. Similar multimodality algorithms are used in ESOSTRATE (NCT02551458) and CELAEC trials to select and monitor patients on non-operative pathways [91]. As demonstrated in preSANO, current modalities have known limitations with suboptimal sensitivity and negative predictive value for detecting residual disease [42]. Although untested in organ-preservation studies, the integration of MRI alongside existing clinical response tools is appealing. Future research should evaluate whether MRI-based criteria can safely increase organ-preservation rates or reduce the incidence of unexpected residual disease at salvage surgery.

### 6.3. Current Clinical Evidence

The clinical rationale for MRgRT also arises from current treatment limitations. The overall incidence of cardiac toxicity is 10.8% (range 5–44%) following oesophageal radiotherapy, mostly occurring within two years [92]. Patients receiving neoadjuvant chemoradiotherapy have a 14.5% increased absolute risk of grade ≥ 3 cardiac events compared to surgery alone, and a higher cardiac dose is consistently associated with poorer overall survival [93,94]. Similarly, survival outcomes are significantly compromised by respiratory complications linked to higher mean lung doses and V20 (volume receiving ≥20 Gy) [95]. Dosimetric studies demonstrate that MR-Linac-based radiotherapy can reduce cardiopulmonary radiation exposure by 26% in mean lung dose and 12% in mean heart dose compared with conventional techniques [96]. Maximum inspiration breath-hold gated MR-Linac can achieve further cardiac sparing for GEJ tumours, reducing mean heart dose from 27.8 Gy to 20.9 Gy [72].

Despite these dosimetric advantages, clinical evidence for MRgRT in oesophageal cancer remains limited to feasibility and early-phase studies. In the R-IDEAL study from the University Medical Center Utrecht, 89% of patients completed planned MR-guided chemoradiotherapy with no acute grade 3+ toxicities; however, median treatment times of 53 min per fraction highlight workflow challenges and limited patient throughput [96]. Emerging workflow optimisation studies and streamlined “ATS-lite” protocols suggest that total session times can be reduced towards 20–30 min, although this is dependent on institutional experience and resources [96,97]. Broader implementation of MR-Linac systems is further constrained by high capital investment, increased staffing requirements, workflow complexity, and limited access to specialised infrastructure and training [34,39,40].

These factors have important health economic implications, particularly for long-course chemoradiotherapy, where online adaptation may be frequent. Current cost-effectiveness analyses are largely extrapolated from other tumour sites and remain highly sensitive to assumptions regarding workflow efficiency and clinical benefit [98]. Accordingly, oesophageal-specific evaluation will require prospective collection of resource utilisation metrics alongside patient-centred outcomes. In the absence of comparative trials demonstrating improvements in clinical outcomes (e.g., hospital admissions, late morbidity, quality of life, or survival), dosimetric gains are viewed as plausible surrogate endpoints supported by cardiopulmonary dose–toxicity relationships [95,99]. Confirmation that these reductions translate into improved clinical outcomes and cost-effectiveness remains necessary. A pragmatic near-term approach is selective implementation in patients most likely to benefit, such as those with high predicted cardiac or pulmonary dose, substantial motion uncertainty, or re-irradiation scenarios.

Recent perioperative trials such as ESOPEC and MATTERHORN have shifted the management of resectable distal and junctional adenocarcinoma toward systemic therapy-based pathways [100,101]. Within this evolving treatment landscape, MRgRT is most clinically relevant in settings where radiotherapy remains central, particularly definitive chemoradiotherapy for squamous cell carcinoma, medically inoperable patients, and emerging response-adapted or organ-preservation strategies. In these contexts, the dosimetric advantages and improved soft-tissue visualisation offered by MRI-Linac systems may support safer and more individualised radiotherapy delivery in selected patients. Key differences between conventional CT/CBCT-guided radiotherapy and MRgRT workflows are summarised in Table 1.

## 7. Conclusions

MRgRT represents an important technical evolution in oesophageal cancer radiotherapy, offering superior soft-tissue contrast, functional imaging, and real-time adaptation. Collectively, early clinical data and dosimetric studies suggest that MRgRT can improve tumour delineation, reduce setup and motion uncertainty, and reduce cardiopulmonary dose without compromising target coverage. These features provide a biological and technologically plausible rationale for more precise, safer, and more personalised radiotherapy.

Nevertheless, current evidence remains limited to small feasibility and dosimetric studies, and no prospective phase II/III trials have yet demonstrated improvements in survival, organ preservation, or patient-reported outcomes. Broader implementation remains constrained by high cost, workflow complexity, MR-specific contraindications, and limited access to MR-Linac technology.

Although recent perioperative systemic trials have reshaped the management of resectable distal and junctional adenocarcinoma towards systemic therapy-focused pathways, radiotherapy remains a key component in the management of oesophageal cancers. Clarifying where MRgRT adds meaningful value within multimodality pathways remains an important area for future investigation.

Future priorities include prospective outcome-focused trials, the standardisation and validation of multiparametric MRI biomarkers, and integration of artificial intelligence to improve efficiency of contouring, motion management, and adaptive workflows. Innovations such as MR-integrated proton therapy may expand the benefits of precision radiotherapy even further as technology matures [102]. With continued technical advancement and clinical validation, MRgRT may evolve from an emerging innovation into a selectively applied component of personalised radiotherapy for oesophageal cancer.

## Figures and Tables

**Table 1:** Comparison of CT/CBCT-Guided Radiotherapy and MR-Guided Adaptive Radiotherapy Workflows in Oesophageal Cancer.

	CT/CBCT-Guided Radiotherapy	MR-Guided Adaptive Radiotherapy
Image guidance and setup	CBCT with limited soft-tissue contrast. Alignment commonly bony or surrogate-based.	Daily MRI with direct tumour and OAR visualisation enabling soft tissue matching.
Motion management	Passive (ITV from 4D-CT). Surrogate-based gating or breath-hold.	Real-time cine-MRI with direct tumour tracking, gating, and drift correction.
Typical CTV→PTV margins	8–10 mm with bony matching. ~5–7 mm with fiducials.	~6 mm in adaptive workflows.
Cardiac and pulmonary dose	Higher mean heart and lung dose, especially for distal/GEJ tumours.	Decrease in mean heart dose ~12–25% and mean lung dose ~20–30% in dosimetric studies.
Adaptive replanning	Offline only. Limited ability to correct anatomical change.	Online daily adaptation based on anatomy of the day.
Fraction treatment time	~10–15 min.	~45–60 min in early implementation; ~20–30 min with streamlined workflows.
Evidence level	Used in standard CRT practice, including phase II/III trials.	Feasibility and dosimetric studies only, no phase II/III outcome data.
Cost and throughput	Lower capital cost, high throughput.	High capital and staffing cost, lower throughput, cost-effectiveness unproven.

## Data Availability

No new data were created or analysed in this study. Data sharing is not applicable to this article.

## Abbreviations

The following abbreviations are used in this manuscript:

ADC	Apparent Diffusion Coefficient
AUC	Area Under the Curve
BOLD-MRI	Blood Oxygen Level-Dependent Magnetic Resonance Imaging
CBCT	Cone-Beam Computed Tomography
CT	Computed Tomography
CTV	Clinical Target Volume
DCE-MRI	Dynamic Contrast-Enhanced Magnetic Resonance Imaging
DWI	Diffusion-Weighted Imaging
EUS	Endoscopic Ultrasound
FDG-PET	Fluorodeoxyglucose Positron Emission Tomography
GEJ	Gastroesophageal Junction
GTV	Gross Tumour Volume
ITV	Internal Target Volume
IVIM	Intravoxel Incoherent Motion
MRI	Magnetic Resonance Imaging
MRgRT	MR-Guided Radiotherapy
MR-Linac	Magnetic Resonance Linear Accelerator
MR-Sim	Magnetic Resonance Simulation
pCR	Pathological Complete Response
PET	Positron Emission Tomography
PTV	Planning Target Volume
r-VIBE	Radial Volumetric Interpolated Breath-Hold Examination
SPIO	Superparamagnetic Iron Oxide
SUV	Standardised Uptake Value
TOLD	Tissue Oxygen Level-Dependent Magnetic Resonance Imaging
